# Avoidable Mortality in Korea 1997–2001: Temporal Trend and its Contribution to All-cause Mortality

**DOI:** 10.3389/ijph.2024.1606825

**Published:** 2024-06-24

**Authors:** Yoolwon Jeong, Sunghyo Seo

**Affiliations:** ^1^ Dankook University Hospital, Cheonan, Republic of Korea; ^2^ Gyeongsang National University Hospital, Jinju, Republic of Korea

**Keywords:** mortality, trends, Republic of Korea, avoidable mortality, premature mortality

## Abstract

**Objective:**

This study analyzed the mortality trends from avoidable causes in Korea from 1997 to 2021, to estimate its contribution to the overall mortality in different subgroups, including. Gender, age, and cause of disease.

**Methods:**

The all-cause and avoidable mortality were presented as a time series plot and average annual percent change. Trend of avoidable mortality was also analyzed by subgroups, disease causes and the percentage attributed to each causes.

**Results:**

The decline in avoidable mortality accounted for 82.6% of all-cause mortality reduction. Preventable mortality showed a more pronounced decline than treatable mortality, explaining 72.3% of the avoidable mortality reduction. In 1997–2001, avoidable death occurred in 72.2% (537,024 cases) of all-cause deaths, which declined to 60.0% (342,979 cases) in 2017–2021. The contribution of avoidable mortality in the decline of all-cause morality was greater in males (83.6%) than in females (79.3%).

**Conclusion:**

The decline in avoidable mortality and its contribution to the all-cause mortality reduction implies general improvement of the population health in Korea. Nevertheless, the heterogenous trend within different subgroups warrants more equitable design and implementation of health services and policies.

## Introduction

Life expectancy in the Republic of Korea (hereafter Korea) has been increasing, presumably owing to the advancement of the healthcare system and the implementation of public health policies [1]. Although the significance of health system performance in population health is increasing, its assessment has not always been straightforward. The concept of avoidable mortality has been used to evaluate the performance of public health policies and healthcare systems in avoiding premature mortality from preventable and treatable causes of death [2, 3]. Avoidable mortality refers to premature deaths from conditions that are considered largely avoidable if appropriate intervention is provided in a timely and effective manner [4]. Avoidable mortality consists of 1) preventable mortality, which is death that can be mainly avoided through public health policies and primary prevention interventions, and 2) treatable mortality, which is death that can be mainly avoided through timely and effective healthcare interventions (e.g., early detection and treatment).

Studies in developed countries have generally demonstrated declines in avoidable mortality [5, 6]. However, patterns and characteristics of such trends, especially with regard to population indicators (e.g., gender, age cohorts), have not been actively explored. Assessing major causes of avoidable deaths is important in identifying areas of excess mortality and examining the influence of health policy and the health system. However, partly due to methodologic barriers, such as varying definitions and classifications of avoidable deaths that researchers have applied over the years, causes of avoidable deaths have been poorly characterized [4–6]. Recently, the Organization for Economic Cooperation and Development (OECD)/Eurostat classification has generally been agreed upon as an international standard list of available mortality. Only a few studies on avoidable mortality have previously been conducted in Korea [1, 7–9], none of which were based on the latest OECD/Eurostat classification (January 2022 version). Previous studies on avoidable mortality in Korea have examined regional disparities [7, 8] and causes of avoidable deaths among age groups [1, 10]. However, the definitions and classification of avoidable mortality used in these studies were variable, hampering domestic and international comparisons.

This study aimed to analyze the mortality trends from avoidable causes using the latest OECD/Eurostat classification (January 2022 version) [4]. We addressed the following research questions:

1. To what extent has all-cause mortality decreased between 1997 and 2021, and how has the decrease in avoidable mortality contributed to this trend?
2. How does this trend in mortality differ among different groups (e.g., gender, age, disease)?

Deaths from preventable and treatable causes were assessed respectively to analyze their contribution to the overall avoidable mortality trend in Korea over a 24-year span from 1997 to 2021.

## Methods

### Data Source and Study Population

The number of deaths by sex and age from 1997 to 2021, based on the International Classification of Diseases (ICD) 10th revision, was obtained from the Micro Data Integrated Service of the Statistics Korea, which is the government agency responsible for national statistics [11]. The data analysis was restricted to deaths under 75 years of age, to comply with the age threshold of premature mortality suggested in the OECD/Eurostat lists of preventable and treatable causes of death (January 2022 version) [4]. Deaths under 5 years of age were excluded from the analysis as numerous previous studies have raised issues in the data reliability of vital statistics stemming from underreporting of infant deaths [8, 10, 12]. Although the results concerning those under 5 years of age are not presented in the results section, their implications in the avoidable mortality study are explored in the discussion section.

### Avoidable Mortality and Standardized Mortality Rate

The definition of avoidable mortality was based on the OECD/Eurostat lists of preventable and treatable causes of death (January 2022 version). The detailed list of preventable and treatable causes of death can be found in: https://www.oecd.org/health/healthsystems/Avoidable-mortality-2019-Joint-OECD-Eurostat-Listpreventable-treatable-causes-of-death.pdf [4]. The OECD/Eurostat list was selected for this study as it more extensively includes diseases with public health significance in Korea (e.g., tuberculosis) and also includes factors related to contacts with health services (e.g., drug adverse events) as compared to other existing classifications. Preventable mortality is defined as “causes of death that can be mainly avoided through effective public health and primary prevention interventions,” and treatable mortality is defined as “causes of death that can be mainly avoided through timely and effective healthcare interventions, including secondary prevention and treatment.” The attribution of causes of mortality to respective categories is based on whether it is prevention or healthcare interventions that predominantly function to reduce death from these causes. While in general, the two categories do not overlap, a 50%–50% allocation was applied in the OECD/Eurostat list when there was no predominance to either category. In this study, this was calculated as 0.5 deaths in preventable and treatable mortality, respectively. For standardization, the direct method was applied by calculating expected deaths using mid-year population by age groups for the 5-year groups. The total population in 2021 was used as the standard population to calculate the standardized mortality rate for all-cause, avoidable, preventable, and treatable deaths.

### Statistical Analysis

The time trend of age-standardized all-cause, avoidable, preventable, and treatable mortality rates per 100,000 population were presented as time series plot by 5-year groups. The number of deaths from avoidable, preventable, and treatable causes, their percentage among all-cause death, as well as its percent point difference between 1997–2001 and 2017–2021, were analyzed by gender and by age groups. Age-standardized mortality by disease causes, their absolute and relative changes from 1997–2001 to 2017–2021, and the percentage attributed to each causes were also assessed. All statistical analyses were performed using the SAS software version 9.4 (SAS Institute Inc., Cary, NC, United States). To further describe the trends in cause-specific avoidable death, average annual percent change (AAPC) and 95% confidence intervals (CI) were calculated using the Joinpoint Regression Programversion 5.0.2 (U.S. National Cancer Institute). We considered *p* values less than .05 to be statistically significant.

## Results

**FIGURE 1:**
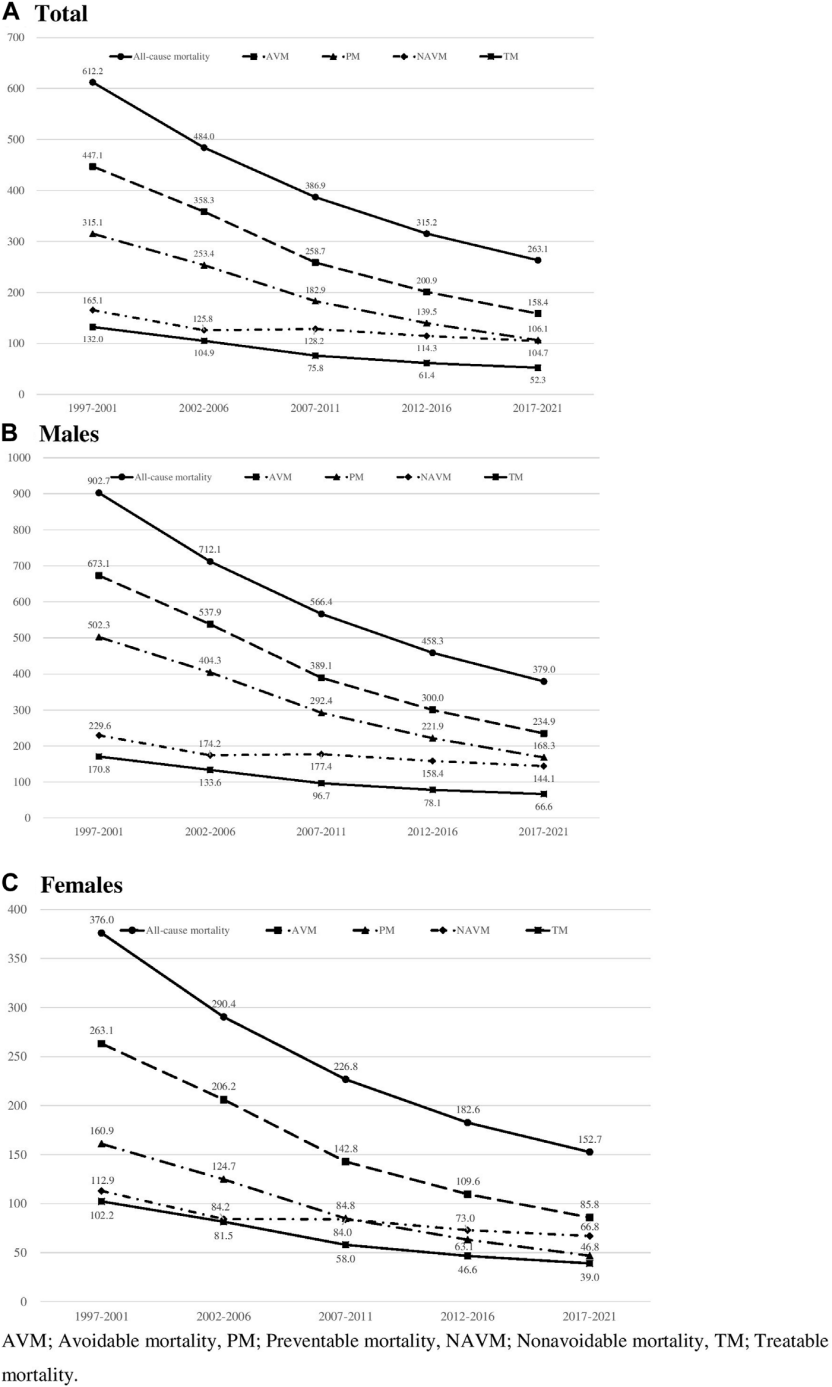
Age standardized all-cause and avoidable mortality rates per 100,000 in all genders, males, and females in Korea, 1997–2021 (South Korea, 2023).

Descriptive characteristics of all deaths in Korea during the study period are presented (Table 1). A total of 3,266,033 deaths occurred from 1997 to 2021. The absolute number of deaths has constantly decreased during the study period from 743,292 in 1997–2001 to 572,020 in 2017–2021. Age-standardized mortality rates per 100,000 population of all-cause mortality, avoidable mortality, and non-avoidable mortality decreased from 1997–2001 to 2017–2021 (Figure 1). However, the decline in avoidable mortality (−64.6%, −288.7/100,000) was more pronounced than in non-avoidable mortality (−36.6%, −60.4/100,000), with a 28.0% excess reduction in avoidable mortality over non-avoidable mortality. In absolute terms, this represents a gain of 85,250 deaths avoided per year during the 2017–2021 period, had the mortality rate of 1997–2001 been maintained. The decline in avoidable mortality accounted for 82.6% of the reduction of all-cause mortality from 1997–2001 to 2017–2021. Among avoidable mortality, preventable mortality showed a higher decline than treatable mortality in relative (−66.3%) and absolute terms (−209.0/100,000), which explained 72.3% of the avoidable mortality reduction.

**TABLE 1:** Descriptive characteristics of all deaths in Korea, 1997–2021 (South Korea, 2023).

	1997–2001	2002–2006	2007–2011	2012–2016	2017–2021
Total (n = 3,266,033)	743,292	689,294	653,354	608,073	572,020
Gender					
Male	496,985	466,159	449,225	424,022	402,235
Female	246,307	223,135	204,129	184,051	169,785
Age group					
5 to 14	8,353	5,810	3,885	2,386	1,885
15 to 24	24,797	14,607	12,540	9,879	8,568
25 to 34	41,931	29,108	26,365	19,958	16,451
35 to 44	84,996	69,085	56,790	46,103	36,945
45 to 54	116,172	118,664	122,214	110,497	93,243
55 to 64	200,684	171,241	151,160	162,742	176,263
65 to 74	266,359	280,779	280,400	256,508	238,665
Cause of death^a^					
Certain infectious and parasitic diseases	19,532	17,097	16,128	13,909	14,740
Neoplasms	219,256	234,937	235,568	228,762	214,922
Diseases of the blood and bloodforming organs and certain disorders involving the immune mechanism	1,589	1,317	1,403	1,405	1,422
Endocrine, nutritional and metabolic diseases	37,036	38,627	29,583	24,443	17,921
Mental and behavioral disorders	13,066	8,572	6,942	6,170	6,386
Diseases of the nervous system	9,314	10,800	11,698	13,525	14,240
Diseases of the eye and adnexa	28	0	7	2	5
Diseases of the ear and mastoid process	9	8	8	5	9
Diseases of the circulatory system	165,260	144,977	116,045	100,510	89,610
Diseases of the respiratory system	30,399	26,148	25,200	28,507	32,885
Diseases of the digestive system	60,874	47,400	37,587	35,109	34,705
Diseases of the skin and subcutaneous tissue	686	538	569	535	614
Diseases of the musculoskeletal system and connective tissue	4,476	3,494	3,179	2,818	2,301
Diseases of the genitourinary system	9,411	9,220	10,901	10,994	10,743
Pregnancy, childbirth and the puerperium	342	294	289	234	169
Certain conditions originating in the perinatal period	0	0	1	0	3
Congenital malformations, deformations and chromosomal abnormalities	839	922	779	699	688
Symptoms, signs and abnormal clinical and la -boratory findings	37,297	22,140	30,859	30,258	34,499
External causes of morbidity and mortality	133,878	122,803	126,608	110,188	96,158

^a^ Cause of death classified according to the International Classification of Diseases 10th revision.

Avoidable mortality decreased in both genders. However, the contribution of avoidable mortality in the decline of all-cause mortality was greater in males (83.6%) than in females (79.3%). In both genders, the contribution of preventable mortality was greater than that of treatable mortality in the decline of all-cause mortality. Among all-cause deaths that occurred during the study period, 2,193,487 cases (67.1%) were death from avoidable causes (Table 2). Among death from avoidable causes, 1,548,907 (70.6% of avoidable death) and 644,579 (29.4% of avoidable death) cases were due to preventable and treatable deaths, respectively. In 1997–2001, death from avoidable causes occurred in 537,024 cases, accounting for 72.2% of all-cause deaths. However, the percentage of avoidable death among all-cause deaths continuously declined during the study period, reaching 60.0% by 2017–2021 (61.6% in males and 56.2% in females). The decline was more pronounced in preventable causes of death (−12.1 percent point difference) than in treatable causes of death (−0.2 percent point difference). This trend was more pronounced in males, as the percentage decreased by 12.7 in preventable causes of death, whereas it slightly increased by 0.4 in treatable causes of death between 1997–2001 and 2017–2021.

**TABLE 2:** Avoidable, preventable, and treatable cause of death in Korea by gender and age group, 1997–2021 (South Korea, 2023).

	Death from avoidable causes (n = 2,193,487)						Death from preventable causes (n = 1,548,907)						Death from treatable causes (n = 644,579)					
	1997 −2001	2002 −2006	2007 −2011	2012 −2016	2017 −2021	PPD	1997 −2001	2002 −2006	2007 −2011	2012 −2016	2017 −2021	PPD	1997 −2001	2002 −2006	2007 −2011	2012 −2016	2017 −2021	PPD
Total^a^	537,024 (72.2)	501,407 (72.7)	428,846 (65.6)	383,231 (63.0)	342,979 (60.0)	−12.3	388,747 (52.3)	358,877 (52.1)	304,771 (46.6)	266,597 (43.8)	229,917 (40.2)	−12.1	148,277 (19.9)	142,531 (20.7)	124,075 (19.0)	116,635 (19.2)	113,063 (19.8)	−0.2
Gender^b^																		
Male	367,151 (73.9)	345,620 (74.1)	302,574 (67.4)	273,455 (64.5)	247,599 (61.6)	−12.3	283,204 (57.0)	264,187 (56.7)	229,718 (51.1)	203,514 (48.0)	177,950 (44.2)	−12.7	83,948 (16.9)	81,434 (17.5)	72,857 (16.2)	69,942 (16.5)	69,649 (17.3)	0.4
Female	169,873 (69.0)	155,787 (69.8)	126,272 (61.9)	109,776 (59.6)	95,380 (56.2)	−12.8	105,544 (42.9)	94,690 (42.4)	75,054 (36.8)	63,083 (34.3)	51,967 (30.6)	−12.2	64,330 (26.1)	61,097 (27.4)	51,219 (25.1)	46,693 (25.4)	43,414 (25.6)	−0.5
Age group^b^																		
5 to 14	5,386 (64.5)	3,561 (61.3)	1,936 (49.8)	1,150 (48.2)	731 (38.8)	−25.7	4,456 (53.3)	2,742 (47.2)	1,424 (36.7)	822 (34.5)	496 (26.3)	−27.1	930 (11.1)	819 (14.1)	512 (13.2)	328 (13.7)	236 (12.5)	1.4
15 to 24	15,560 (62.7)	7,527 (51.5)	5,104 (40.7)	3,879 (39.3)	2,631 (30.7)	−32.0	13,831 (55.8)	6,267 (42.9)	4,255 (33.9)	3,136 (31.7)	1,997 (23.3)	−32.5	1,730 (7.0)	1,260 (8.6)	849 (6.8)	744 (7.5)	634 (7.4)	0.4
25 to 34	26,310 (62.7)	15,734 (54.1)	10,531 (39.9)	7,696 (38.6)	5,677 (34.5)	−28.2	21,951 (52.3)	12,367 (42.5)	8,054 (30.5)	5,704 (28.6)	3,984 (24.2)	−28.1	4,360 (10.4)	3,367 (11.6)	2,478 (9.4)	1,992 (10.0)	1,694 (10.3)	−0.1
35 to 44	59,566 (70.1)	46,302 (67.0)	32,343 (57.0)	24,111 (52.3)	18,118 (49.0)	−21.0	47,499 (55.9)	35,807 (51.8)	24,374 (42.9)	17,195 (37.3)	12,262 (33.2)	−22.7	12,068 (14.2)	10,495 (15.2)	7,970 (14.0)	6,917 (15.0)	5,857 (15.9)	1.7
45 to 54	86,541 (74.5)	87,094 (73.4)	80,945 (66.2)	69,733 (63.1)	55,432 (59.4)	−15.0	66,479 (57.2)	66,541 (56.1)	60,626 (49.6)	50,530 (45.7)	37,984 (40.7)	−16.5	20,062 (17.3)	20,554 (17.3)	20,319 (16.6)	19,203 (17.4)	17,449 (18.7)	1.4
55 to 64	152,224 (75.9)	129,653 (75.7)	104,472 (69.1)	107,381 (66.0)	110,908 (62.9)	−12.9	110,310 (55.0)	94,079 (54.9)	75,713 (50.1)	76,619 (47.1)	76,199 (43.2)	−11.7	41,914 (20.9)	35,575 (20.8)	28,759 (19.0)	30,762 (18.9)	34,710 (19.7)	−1.2
65 to 74	191,437 (71.9)	211,536 (75.3)	193,515 (69.0)	169,281 (66.0)	149,482 (62.6)	−9.2	124,223 (46.6)	141,075 (50.2)	130,326 (46.5)	112,592 (43.9)	96,997 (40.6)	−6.0	67,215 (25.2)	70,462 (25.1)	63,189 (22.5)	56,690 (22.1)	52,485 (22.0)	−3.2

^a^ Percentage among all-cause death under 75 years of age.

^b^ Percentage among all-cause death in respective subgroups, PPD; Percent point difference between 1997–2001 and 2017–2021.

In age group analysis, the percentage of death from avoidable causes among all-cause deaths was lowest (62.7%) in age groups (1) 15 to 24 and (2) 25 to 34 in 1997–2001. The decline in percent point difference throughout the study period was also highest in these groups, reaching 30.7% and 34.5%, respectively, by 2017–2021. On the contrary, the percentage of death from avoidable causes among all-cause deaths was the highest in the age group of 55–64 (75.9%) in 1997–2001, which decreased to 62.9% by 2017–2021. In all age groups, the number of death from preventable causes was higher than that of treatable causes, and their percentage in all-cause death declined. However, the trend was variable in deaths from treatable causes, decreasing in certain age groups (55–64 and 65–74) and increasing in others. The greatest decline was observed in the oldest age group (65–74).

Analysis of mortality trends from each avoidable cause of death reveals that in 1997–2001, mortality was highest in neoplasms (145.0/100,000), followed by diseases of the circulatory system (128.3/100,000) and injuries (55.1/100,000) (Table 3). This trend was consistent in 2017–2021, with neoplasms (68.2/100,000), diseases of the circulatory system (31.2/100,000), and injuries (15.0/100,000) as the top three leading causes of avoidable death, albeit with lower mortality rates. The decline in mortality rates was pronounced and statistically significant in injuries (AAPC = −6.6), endocrine and metabolic diseases (AAPC = −6.5), and diseases of the circulatory system (AAPC = −6.3). In males, mortality was highest in neoplasms (229.8/100,000), followed by diseases of the circulatory system (169.4/100,000) and injuries (86.2/100,000) in 1997–2001. This trend was consistent in 2017–2021, with neoplasms (96.7/100,000), diseases of the circulatory system (46.1/100,000), and injuries (24.0/100,000) as the top three leading causes of avoidable death. The decline in mortality rates was pronounced and statistically significant in injuries (AAPC = −6.5), endocrine and metabolic diseases (AAPC = −5.9), and diseases of the circulatory system (AAPC = −5.8). In females, mortality was highest in diseases of the circulatory system (96.0/100,000) followed by neoplasms (79.4/100,000) and injuries (26.5/100,000) in 1997–2001. Mortality from diseases of the circulatory system declined by −79.1/100,000 (AAPC = −7.9), leaving neoplasms as the top cause of death in 2017–2021. Mortality due to endocrine and metabolic diseases showed the greatest decline (AAPC = −8.3). While the top three leading causes of avoidable deaths were identical in both genders (neoplasm, diseases of the circulatory system, and injuries), the following, fourth cause varied between genders. In males, alcohol- and drug-related death was the fourth most common cause of avoidable deaths in all period groups from 1997 to 2016, and attributable to 9.96% of all avoidable deaths in 2017–2021. As for females, endocrine and metabolic diseases have been the fourth most common cause of avoidable death throughout the years. However, the rate has constantly declined to rank as the sixth most common cause of avoidable death (4.0/100,000), after diseases of the respiratory system (6.0/100,000) and alcohol- and drug-related death (5.2/100,000) in 2017–2021. This decreasing trend in most cause-specific avoidable mortality is consistent with decreasing trends in total avoidable mortality (AAPC = −4.75, 95% CI: −4.87, −4.66), avoidable mortality in males (AAPC = −4.84, 95% CI: −4.95, −4.76) and females (AAPC = −5.04, 95% CI: −5.2, −4.93) (Supplementary Figure S1).

**TABLE 3:** Trend of cause-specific avoidable death in Korea, 1997–2021 (South Korea, 2023).

Causes of disease	1997–2001		2002–2006		2007–2011		2012–2016		2017–2021		Change		
	SMR	%	SMR	%	SMR	%	SMR	%	SMR	%	Ab	Rl	AAPC
Total													
Adverse effects of medical and surgical care	0.2	0.04	0.3	0.07	0.2	0.08	0.2	0.09	0.1	0.09	−0.1	−50.0	0.5
Alcohol-related and drug-related deaths	40.4	9.03	29.4	8.20	18.7	7.24	15.3	7.64	14.2	8.98	−26.2	−64.9	−4.8*
Congenital malformations	0.2	0.05	0.3	0.07	0.2	0.07	0.1	0.06	0.1	0.07	−0.1	−50.0	−3.4*
Diseases of the circulatory system	128.3	28.70	98.7	27.55	60.5	23.36	41.4	20.61	31.2	19.71	−97.1	−75.7	−6.3*
Diseases of the digestive system	3.2	0.72	1.8	0.49	1.8	0.69	1.5	0.74	1.2	0.75	−2.0	−62.5	−5.4*
Diseases of the genitourinary system	1.3	0.28	0.6	0.16	0.7	0.28	0.7	0.33	0.7	0.44	−0.6	−46.2	−3.3*
Diseases of the nervous system	1.0	0.23	1.1	0.32	0.8	0.31	0.7	0.34	0.6	0.40	−0.4	−40.0	−2.5*
Diseases of the respiratory system	23.4	5.24	17.8	4.97	13.7	5.28	13.2	6.59	13.3	8.39	−10.1	−43.2	−2.6*
Endocrine and metabolic	33.0	7.39	28.6	7.97	17.5	6.77	11.9	5.95	7.1	4.50	−25.9	−78.5	−6.5*
Infectious diseases	15.9	3.56	11.5	3.22	9.2	3.57	6.9	3.42	5.5	3.44	−10.4	−65.4	−5.2*
Injuries	55.1	12.32	37.3	10.41	27.8	10.75	21.3	10.60	15.0	9.50	−40.1	−72.8	−6.6*
Neoplasms	145.0	32.42	131.0	36.56	107.6	41.57	87.6	43.59	68.2	43.07	−76.8	−53.0	−3.5*
Pregnancy, childbirth, perinatal period	0.1	0.02	0.1	0.03	0.1	0.04	0.1	0.04	0.1	0.04	0.0	0.0	−3.1*
Male													
Adverse effects of medical and surgical care	0.2	0.03	0.3	0.05	0.2	0.05	0.2	0.06	0.2	0.08	0.0	−9.4	−1.6
Alcohol-related and drug-related deaths	72.2	10.72	52.0	9.67	33.0	8.48	26.3	8.75	23.4	9.96	−48.8	−67.6	−5.2*
Congenital malformations	0.2	0.03	0.2	0.04	0.2	0.04	0.1	0.04	0.1	0.05	−0.1	−46.1	−3.3*
Diseases of the circulatory system	169.4	25.16	131.9	24.52	84.1	21.62	59.4	19.79	46.1	19.64	−123.2	−72.8	−5.8*
Diseases of the digestive system	5.1	0.75	2.6	0.48	2.6	0.68	2.2	0.74	1.8	0.77	−3.3	−64.4	−5.7*
Diseases of the genitourinary system	1.8	0.27	0.8	0.15	1.0	0.26	0.9	0.31	1.0	0.43	−0.8	−44.4	−3.4*
Diseases of the nervous system	1.4	0.20	1.6	0.30	1.1	0.28	1.0	0.32	0.9	0.38	−0.5	−34.3	−2.2*
Diseases of the respiratory system	39.1	5.82	30.6	5.70	22.7	5.84	21.4	7.13	21.2	9.02	−18.0	−45.9	−2.7*
Endocrine and metabolic	41.4	6.15	36.7	6.82	23.7	6.09	16.7	5.55	10.4	4.43	−31.0	−74.9	−5.9*
Infectious diseases	26.4	3.92	18.2	3.38	13.6	3.48	9.9	3.32	7.7	3.28	−18.7	−70.8	−6.0*
Injuries	86.2	12.80	58.8	10.93	44.3	11.40	33.7	11.23	24.0	10.22	−62.2	−72.1	−6.5*
Neoplasms	229.8	34.14	204.2	37.96	162.5	41.78	128.2	42.75	96.7	41.15	−133.2	−57.9	−4.1*
Female	-	-	-	-	-	-	-	-	-	-	-	-	
Adverse effects of medical and surgical care	0.1	0.06	0.2	0.10	0.2	0.14	0.2	0.17	0.1	0.15	0.0	−11.4	0.3
Alcohol-related and drug-related deaths	12.1	4.60	8.4	4.09	5.2	3.61	4.8	4.36	5.2	6.04	−6.9	−57.2	−4.0*
Congenital malformations	0.2	0.09	0.3	0.12	0.2	0.12	0.1	0.12	0.1	0.11	−0.1	−59.7	−4.1*
Diseases of the circulatory system	96.0	36.49	70.8	34.33	39.5	27.65	24.8	22.60	17.0	19.75	−79.1	−82.3	−7.9*
Diseases of the digestive system	1.8	0.67	1.1	0.51	1.0	0.68	0.8	0.72	0.6	0.71	−1.2	−65.4	−5.6*
Diseases of the genitourinary system	0.8	0.32	0.4	0.19	0.5	0.32	0.4	0.36	0.4	0.48	−0.4	−50.4	−3.9*
Diseases of the nervous system	0.7	0.26	0.7	0.35	0.5	0.36	0.4	0.37	0.4	0.44	−0.3	−45.4	−3.7*
Diseases of the respiratory system	11.9	4.53	8.0	3.89	6.2	4.32	6.1	5.54	6.0	7.04	−5.9	−49.4	−2.9*
Endocrine and metabolic	25.9	9.83	21.4	10.36	12.0	8.38	7.6	6.94	4.0	4.66	−21.9	−84.5	−8.3*
Infectious diseases	7.4	2.83	5.8	2.84	5.3	3.69	4.0	3.63	3.3	3.87	−4.1	−55.4	−3.9*
Injuries	26.5	10.06	17.4	8.43	12.1	8.45	9.3	8.47	6.2	7.20	−20.3	−76.6	−7.1*
Neoplasms	79.4	30.18	71.5	34.69	60.2	42.13	51.0	46.56	41.8	48.70	−37.6	−47.3	−3.1*
Pregnancy, childbirth, perinatal period	0.2	0.09	0.2	0.10	0.2	0.15	0.2	0.17	0.2	0.18	−0.1	−34.5	−2.6*

^a^ % percentage of avoidable mortality attributed to each cause group, SMR; Age-standardized mortality rate per 100,000 population, Ab; Absolute change, Rl; Relative change, AAPC; Average Annual Percentage Change. *Indicates statistical significance <0.05.

## Discussion

During the study period of 1997–2021, the decreasing trend of avoidable mortality (by −64.6%) was more pronounced than non-avoidable mortality (by −36.6%), accounting for 82.6% of the reduction of all-cause mortality, suggesting a general improvement of population health in Korea. A similar trend was observed in previous domestic studies, which estimated a decrease in avoidable mortality by −62% [9] and −65% [7] during the study period of 21–24 years, respectively. Similarly, a decrease in avoidable mortality explained 87.4% of all-cause mortality reductions in 2001–2020 in another domestic study [8]. Numerous international studies revealed a similar result with avoidable mortality decreasing more prominently than its non-avoidable counterpart [5, 6, 13–17]. An exception to this trend is shown in a recent study which revealed that an increase in avoidable mortality in 2021 was mainly due to death from Coronavirus Disease 2019 (COVID-19) [18]. In this study, percentage of death from Coronavirus Disease 2019 as to the total cause of death was less than 1% in 2017–2021. Although we speculate that its impact to the overall avoidable mortality during the 24-year period may not be significant, further study is warranted to explore the delayed and accumulated impact of Coronavirus Disease 2019 on the avoidable mortality in Korea.

Although direct comparison requires caution, 72.2% of all deaths were considered avoidable in this study in 1997–2001, which was higher than approximately 50% of Australia in 2001 [6] and Canada in 1999 [15] and more comparable to 70% of New Zealand in 1997 [13]. More recent comparison in 2019 presents that the avoidable mortality in Korea has since dramatically decreased to levels well below the average of OECD countries in both preventable (76% of the OECD average) and treatable causes (57.5% of the OECD average) [19]. Also in this study, the decrease in preventable mortality was more pronounced than treatable mortality and explained 72.3% of the total avoidable mortality reduction. Although the design of this study hampers any assumptions on causal relationships, it adds to the body of evidence that effective health promotion and primary prevention interventions may result in the decrease in preventive mortality [1, 6, 9, 14]. Health promotion usually addresses behavioral risk factors such as tobacco use, diet, obesity, physical inactivity, alcohol consumption, injury prevention, and drug abuse control which targets the community at large. Primary prevention includes consultation and information provision on health risks and clinical preventive services such as immunization. Evidence suggests that health promotion and primary prevention, which are usually population-delivered large-scale interventions, are more cost-effective than waiting until the disease has occurred to intervene curatively on an individual basis [20–22]. Much of the gain in life expectancy over the 20th century is now well known to be attributed to population-based interventions such as safe water and food, tobacco cessation programs, immunizations, etc. [23].

On the other hand, it is interesting to note that compared to treatable mortality, preventive mortality tends to be more prone to inequalities and affects more heterogeneously between groups. In this study, mortality from preventable causes was higher in males than in females. The difference in mortality between genders was higher in preventive than in treatable mortality. This is relevant in previous studies, in which mortality from preventable causes was higher than treatable causes in males than females [7, 13, 24], in aboriginal than non-aboriginal people [13, 25], and in immigrants than in non-immigrants [26]. Similarly in these studies, the difference in mortality between groups was higher in preventive than treatable mortality, suggesting that their effect could be rather disproportionate among different population groups and regions [13].

In Korea, a series of national-level efforts on health promotion and disease prevention have been rolled out during the past several decades. The establishment of the National Health Promotion Act and the National Health Promotion Fund in 1995 was the *de facto* cornerstone of coordinated efforts for health promotion and disease prevention at the national level [7]. The National Health Promotion Act stipulated that the state and local governments should put forth systematic efforts to promote healthy lifestyles and behaviors, create an enabling environment, and provide services regarding disease prevention. The National Health Promotion Fund, which was created partly through revenues from tobacco taxes, allowed more sustainable budgetary allocations to various health promotion programs such as tobacco cessation, national immunization programs, etc. [8]. A national-level, multi-sectoral 5-year strategy on health promotion and disease prevention (Health Plan) was first developed in 2002, with its most recent version (Health Plan 2030) published in 2021. Framework Act on Health and Medical Services (2000) and Public Health and Medical Services Act (2000) underscored the roles and responsibilities of the state and the citizens in health promotion and disease prevention. Disease and topic-specific legislations, e.g., Cancer Control Act (2003), Dental Health Act (2000), and Mother and Child Health Act (1973) among others, were also enacted. Such national-level efforts culminated in the improvement of numerous measures, such as smoking cessation rate in adults and adolescents, the prevalence of adherence to healthy diet plans, and high-risk drinking in males, in 2020 [27].

However, it is worth noting that the current smoking rate, alcohol consumption rate, and high-risk alcohol drinking did not improve or have even worsened among females in Korea [28, 29]. The current smoking rate in adult females has been stable for the past 20 years and even slightly increased in adolescents. The rate of physical activity in females has been 30%–40% lower than in males during the past decade [27–29]. Such gender disparity in health behaviors may explain the more pronounced decline in preventable mortality in males revealed in this study. Analysis of health behaviors in domestic studies revealed that further disparities and inequities exist between social determinants such as gender, region, and income [27]. These findings support our speculation that the effect of health promotion policies and programs may be heterogeneous among groups. To narrow the discrepancies, health promotion policies should consider population characteristics, local context, needs, and values of different groups within the community. In Korea, the Regional Public Health Act (1996) enabled authorities and responsibilities of the local governments to plan and provide health promotion programs within their jurisdictions, taking into account the local context and priorities. More policy effort is warranted to tailor to different population groups and address the gaps in positive health behaviors.

The age group analysis in this study and previous studies [13, 16] revealed that avoidable mortality is higher in middle and old age, in both absolute and relative terms. In age groups above 35 years, avoidable deaths accounted for more than 70% of all deaths. However, it was in younger age groups that avoidable mortality showed a more pronounced decline over the study period. This trend was more dramatic in preventable mortality than in treatable mortality, which was in line with the results of an existing study [30]. As previous studies hints that there may be strong age and cohort effects on avoidable mortality trends [8], such different trends in different age groups may be due to a mixed effect of cohort characteristics and other environmental factors, although further study is warranted to explore any age-cohort-period effect on avoidable mortality. Deaths in the under 5 years group (data not presented) showed a very different trend compared to other age groups, with a much smaller decline in preventive deaths (−11.9 Percent Point Difference, PPD) and a higher increase in treatable deaths (+7.6 PPD). There have been numerous speculations on the possibility of underreporting of infant deaths and resulting issues in data reliability of death certificates before the 21st century in Korea [10, 12], which may have resulted in a decrease in preventable deaths to appear smaller and an increase in treatable deaths to appear higher than the actual picture.

Neoplasms, diseases of the circulatory system, and injuries were the top three leading causes of avoidable death throughout the study period, albeit with a much lower mortality rate at the end of the study period. Diseases of the circulatory system contributed most to the decline in avoidable mortality, which was similar to results from previous studies in other countries [6, 13, 14]. In Korea, the National Health Screening Program, which has been running since the 1980s, was reorganized in 2009 to focus on early detection of cardiovascular diseases. Public health centers, 258 in number as of 2023, rolled out community-based hypertension and diabetes registry program in 2007, through which patients were enrolled and empowered. The designation of Regional Cardiocerebrovascular Centers in major tertiary hospitals started in 2008, enabling treatment and intervention within the golden hour. The National Strategy on Cardio-cerebrovascular Diseases was first developed in 2006, with its fourth version published in 2023. The Act on the Prevention and Management of Cardio-cerebrovascular Diseases was enacted (2017). Efforts to control cancer were even more comprehensive, including legislation and national strategies (The Cancer Control Act, 2003, National Cancer Control Plans), primary preventive measures at community levels, early detection through the National Cancer Screening Program which was expanded to cover the entire population in 2004, enhancing accessibility to treatment and medication through the National Health Insurance and other financial mechanisms (e.g., Cancer Patient Financial Aid System, 2002, Financial support system for Catastrophic Health Expenditure, 2013), survivorship supports and end-of-life care, national cancer registries and statistics, and establishment of governance structures (e.g., National Cancer Center, Regional Cancer Centers, National Cancer Control Institute).

Policy efforts regarding injury prevention have been multi-sectoral, involving the ministries of health, education, rural development, Road Traffic Authority, National Fire Agency, Occupational Safety and Health Agency, and School Safety Federation, among others. In the health sector, national-level strategies have generally been developed within the frameworks of the Health Plan 2010, 2020, and 2030 [27], covering injury surveillance, addressing behavioral risk factors in safety, and establishing safe environments. A multi-sectoral injury surveillance program was established, through which it was revealed that death from injury has decreased from 61.7 per 100,000 in 2008 to 54.7 per 100,00 in 2018, possibly attributable to a pronounced decline in road traffic injuries, reaching the policy goals of the Health Plan [27]. This decline in injuries, especially in road traffic accidents, is quite universal in developed countries [5, 14, 15]. Despite such achievements, falls (+24%), intoxication (+17%), and burns (+4%) have increased over the past decade [27]. In this study, injuries and alcohol- and drug-related deaths were the third and fourth most common cause of death in males, together accounting for about 20% of all avoidable deaths, warranting continued policy efforts to prevent injury [13].

This study has several limitations. Intrinsic to the nature of avoidable mortality, absolute levels of avoidable mortality are not readily comparable between studies due to the different study periods and disease classifications. In addition, the observed changes in avoidable mortality may be attributable to factors outside the healthcare system, and therefore speculations in this study do not present any direct association or causal relationships between health policies and medical interventions with avoidable mortality trends. Another limitation is that the cause of death classifications may not be precise or consistent over the years, possibly owing to changes in the methods in death registrations and co-morbidities in the elderly.

The analysis of the avoidable mortality in Korea from 1997 to 2021 confirmed that deaths from avoidable causes have declined, accounting for the majority of the reduction in all-cause mortality. The decrease in preventable mortality was more pronounced than treatable mortality. Neoplasms, diseases of the circulatory system, and injuries were constantly the top three leading causes of avoidable death, with alcohol- and drug-related death and respiratory diseases as the fourth most common cause of avoidable deaths in males and females, respectively. Improvements in avoidable mortality were disproportionate among different population groups, implying that future policy efforts should tailor to different population groups and address the gaps in social determinants.

## Data Availability

The datasets supporting the conclusions of this article are opensource information made freely available in the public domain by the Statistics Korea (https://kosis.kr). Data underlying this article will be shared on reasonable request by the corresponding author.
