# Supplementary material for: Avoidable Mortality in Korea 1997–2001: Temporal Trend and its Contribution to All-cause Mortality
Source: Int J Public Health. 2024 Jun 24;69:1606825. doi: 10.3389/ijph.2024.1606825 (PMC11228162; doi:10.3389/ijph.2024.1606825)
Supplement: Supplementary file 1 [file DataSheet1.doc]

| **(A)Avoidable mortality**  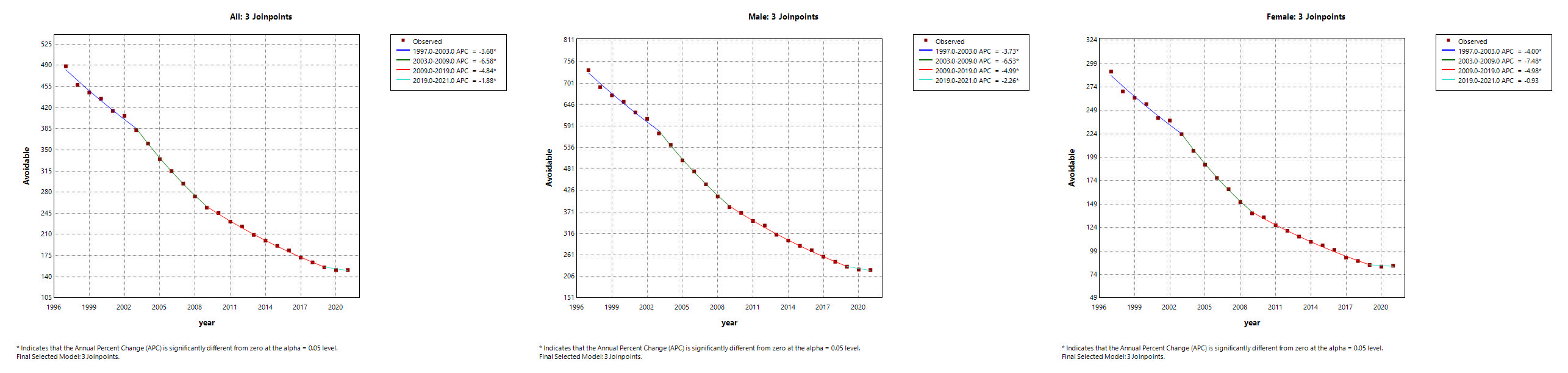 |
| --- |
| **(B)Preventable mortality**  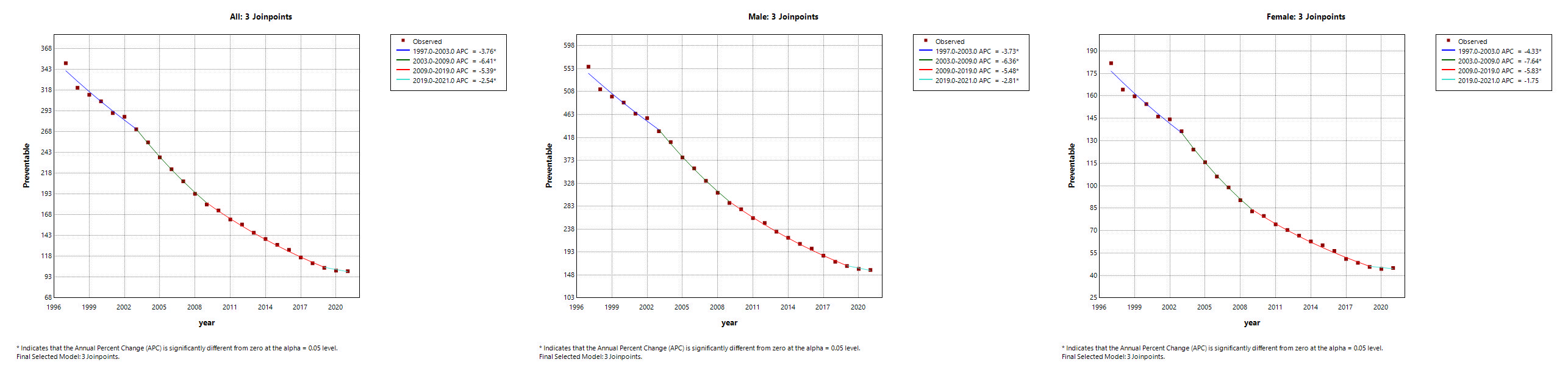  **(C) Treatable mortality**  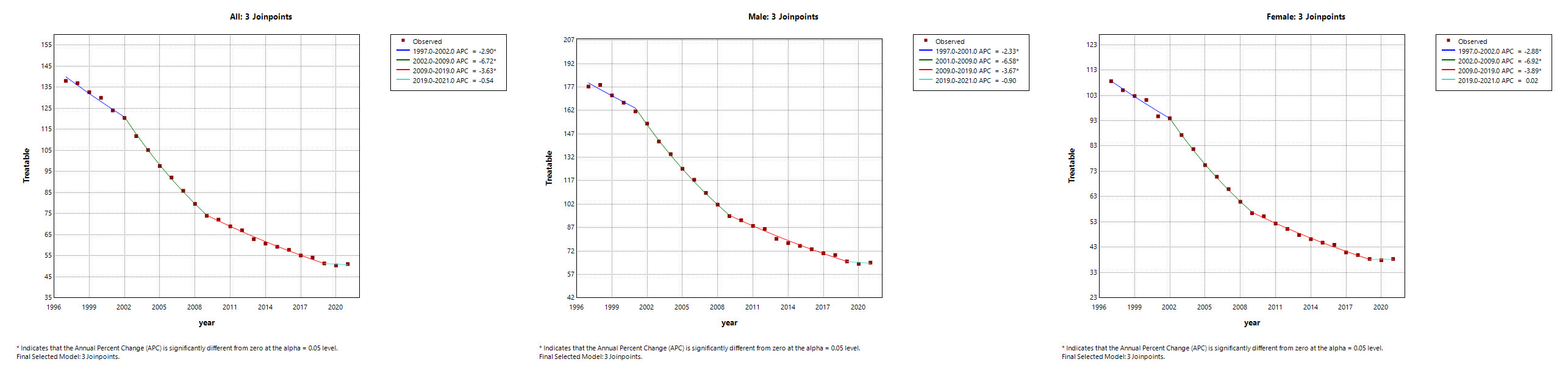 |
| APC; Annual percent change.  **Supplementary Figure 1. Joinpoint regression analysis of the age-standardized avoidable mortality in Korea (South Korea, 2023)** |
|  |
